# Clinical outcomes of endovascular therapy strategy based on thrombus location for acute superior mesenteric artery occlusion

**DOI:** 10.1007/s11604-025-01914-2

**Published:** 2025-11-26

**Authors:** Tatsuo Ueda, Fumie Sugihara, Hidemasa Saito, Sayaka Shirai, Ryutaro Fujitsuna, Taiga Matsumoto, Misa Iwasaki, Shoji Yokobori, Hiroshi Yoshida, Hiromitsu Hayashi, Shin-ichiro Kumita

**Affiliations:** 1 Department of Radiology, Nippon Medical School Hospital, 1-1-5 Sendagi, Bunkyo-ku, Tokyo, 113-8603 Japan; 2 Department of Emergency and Critical Care Medicine, Nippon Medical School Hospital, 1-1-5 Sendagi, Bunkyo-ku, Tokyo, 113-8603 Japan; 3 Department of Gastrointestinal and Hepato-Biliary-Pancreatic Surgery, Nippon Medical School Hospital, 1-1-5 Sendagi, Bunkyo-ku, Tokyo, 113-8603 Japan

**Keywords:** Acute superior mesenteric artery occlusion, Endovascular therapy, Aspiration embolectomy, Antegrade stenting, Local thrombolysis

## Abstract

**Purpose:**

To evaluate the short-term outcomes of patients with acute superior mesenteric artery occlusion who underwent endovascular therapy using different endovascular techniques based on thrombus location.

**Materials and methods:**

This single-center retrospective observational study included consecutive patients with acute superior mesenteric artery occlusion who underwent endovascular therapy using a single or combined approach, including aspiration embolectomy, local thrombolysis, antegrade stenting, and balloon angioplasty, with or without necrotic bowel resection, between January 2007 and December 2024. The thrombus location was classified as proximal, middle, and peripheral based on angiographic findings, and endovascular therapy strategies were selected based on thrombus location. The initial endovascular technique was selected as follows: aspiration embolectomy or antegrade stenting for proximal thrombus, aspiration embolectomy for middle thrombus, and local thrombolysis for peripheral thrombus. Outcomes assessed included technical success (reperfusion of blood flow in the primary occluded superior mesenteric artery segment targeted by endovascular therapy), procedure-related adverse events, bowel necrosis, bowel resection, all-cause 30-day mortality, and acute superior mesenteric artery occlusion-related 30-day mortality.

**Results:**

Thirty-three consecutive patients (mean age 76.0 ± 8.9 years; 20 male) were included. The technical success rate was 100%. Procedure-related adverse events occurred in 15.2% patients (four arterial injuries and one distal embolization). Bowel necrosis was observed in 36.4% patients (n = 12), with 33.3% (n = 11) requiring necrotic bowel resection. All bowel necrosis cases were associated with peripheral thrombus. All-cause 30-day mortality and acute superior mesenteric artery occlusion-related 30-day mortality rates were 15.8% (n = 5) and 9.3% (n = 3), respectively. Extensive thrombus involving the peripheral region was significantly associated with bowel necrosis (*P* < 0.05). All acute superior mesenteric artery occlusion-related deaths were linked to extensive bowel necrosis.

**Conclusions:**

Tailored endovascular strategies based on thrombus location may contribute to favorable clinical outcomes in patients with acute superior mesenteric artery occlusion.

## Secondary abstract

Endovascular therapy (EVT) based on thrombus location for acute superior mesenteric artery occlusion demonstrated *favorable* success rates/lower 30-day mortality. Peripheral thrombus was strongly associated with bowel necrosis; extensive necrosis correlated with higher mortality.

## Introduction

The primary treatment for acute superior mesenteric artery occlusion (ASMAO) is revascularization of viable bowel, often combined with resection of necrotic segments [1]. Five nonrandomized controlled trials showed that bowel resection and 30-day mortality rates are lower with endovascular therapy (EVT) than with open surgery [2–6]. Current guidelines therefore recommend endovascular revascularization as the first-line treatment [1]. Moreover, recent reviews have confirmed its effectiveness as a minimally invasive option [7]. Various endovascular techniques, including aspiration [8–11], thrombolysis [12, 13], stenting [10], and balloon angioplasty [14], are available for ASMAO. Although a combination of these techniques is often performed, selection varies by availability, institution, and interventionalist expertise, with limited evidence regarding optimal selection [15]. Additionally, the appropriate technique may depend on the thrombus location.

Precise localization of vessel occlusion is essential before revascularization, as treatment methods vary, with stenting preferred for proximal lesions and thrombectomy for middle lesions [16–18]. However, evidence on EVT strategy based on thrombus location in ASMAO remains limited [1, 15]. The aim of this study was to evaluate the short-term outcomes of patients with ASMAO undergoing EVT using various endovascular techniques based on thrombus location.

## Materials and methods

### Patients

All procedures involving human participants were performed in accordance with the ethical standards of the institutional and/or national research committee and the 1964 Helsinki Declaration and its amendments or comparable ethical standards. This study was approved by the Institutional Review Board of our institution. Written informed consent was obtained from all patients prior to treatment.

This was a single-center retrospective observational study. The medical record system and official computed tomography (CT) imaging reports from our hospital between January 2007 and December 2024 were reviewed to identify ASMAO cases. Treatment decisions were made by emergency physicians or gastrointestinal surgeons based on bowel viability assessment through CT, laboratory, and clinical findings. Laparotomy with necrotic bowel resection was performed if signs of peritonitis or suspected intestinal infarction were present, unless a palliative approach was selected. Following the laparotomy, residual thrombus was evaluated by angiogram or CT imaging. If residual thrombus was identified after laparotomy, revascularization with EVT was performed. Conversely, if the bowel was deemed salvageable based on the initial bowel viability assessment, EVT was the primary treatment. In cases of residual thrombus post-revascularization, a second-look laparotomy was performed to reassess bowel viability.

From all patients with ASMAO, consecutive patients who underwent EVT using a single technique or a combination of multiple endovascular techniques, with or without necrotic bowel resection, were included in this study. Exclusion criteria were as follows: (1) ASMAO secondary to dissection, (2) patients unable to undergo revascularization owing to critical general condition, and (3) patients treated with open surgery or medical treatment without EVT.

### Classification of thrombus location

**Fig. 1:**
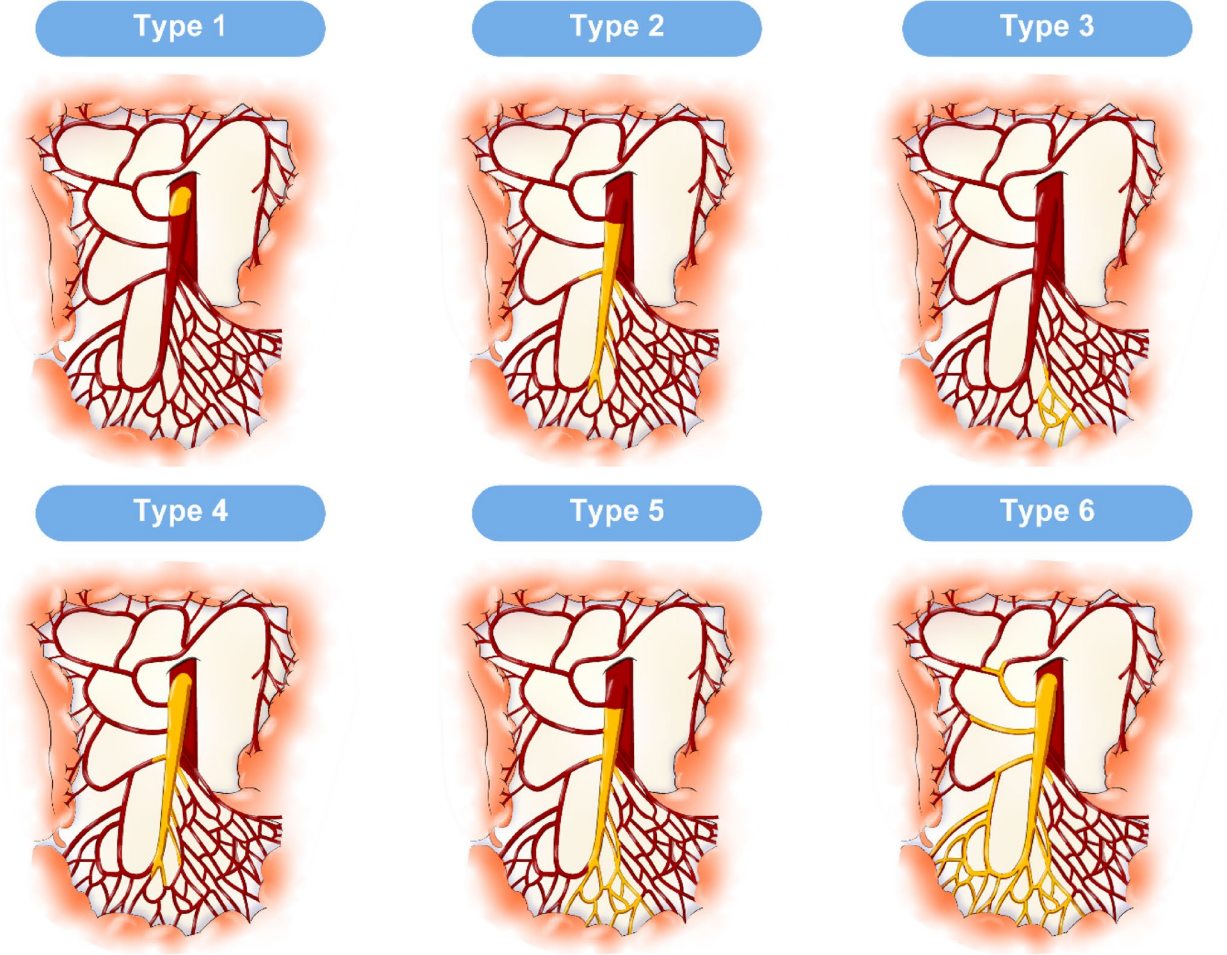
Classification of ASMAO based on thrombus location. **a** Type 1, proximal region; **b** Type 2, middle region; **c** Type 3, peripheral region; **d** Type 4, proximal to middle; **e** Type 5, middle to peripheral; **f** Type 6, proximal to peripheral. *ASMAO* acute superior mesenteric artery occlusion

Thrombus location was classified based on angiographic findings (Fig. 1). The proximal type was defined as a thrombus from the origin of the superior mesenteric artery (SMA) to SMA proximal to the middle colic artery branch. The middle type was defined as a thrombus located from SMA distal to the middle colic artery branch to the first-order SMA branch. The peripheral type was defined as a thrombus distal to the first-order SMA branch. In this study, the first-order SMA branches were defined as the primary jejunal, ileal, ileocolic, and colic branches arising directly from the SMA trunk. Within this classification, occlusion of a first-order SMA branch arising proximal to the middle colic artery was also defined as the peripheral type, provided that the main trunk of the SMA itself was not occluded. Based on this classification, six thrombus patterns were identified: Type 1, proximal region; Type 2, middle region; Type 3, peripheral region; Type 4, proximal to middle; Type 5, middle to peripheral; and Type 6, proximal to peripheral. To facilitate comparison with prior literature, we additionally performed a sensitivity analysis with the six types collapsed into the three conventional groups: proximal (Types 1, 4, and 6), middle (Types 2 and 5), and distal (Type 3) [16].

### Endovascular techniques

EVT was performed in all patients via the common femoral artery using a 4-Fr sheath (Supersheath; Medikit, Tokyo, Japan) under local or general anesthesia. Heparin (5000 IU) was administered intra-arterially, followed by 1000 IU every 1 h to maintain the activated clotting time (ACT) within the range of 200–300 s. After diagnostic arteriography, a 4-Fr catheter (C-MRT; Medikit, Tokyo, Japan) was advanced to the distal side of SMA with the aid of a 0.035-inch guidewire (Radifocus; Terumo, Tokyo, Japan), which was exchanged for a 0.035-inch stiff guidewire (Amplatz Super Stiff, Boston Scientific, Natick, MA) or a 0.035-inch half-stiff guidewire (Radifocus; Terumo, Tokyo, Japan) in order to exchange the sheath for a 6Fr guiding sheath (Destination; Terumo, Tokyo, Japan). EVT was then performed using either a single endovascular technique or a combination of techniques, including aspiration embolectomy, local thrombolysis, antegrade stenting, and balloon angioplasty, through the guiding sheath.

Manual aspiration embolectomy was performed using a 6-Fr guiding catheter (Launcher; Medtronic, Minneapolis, MN, USA) or a 6-Fr aspiration catheter (AspirareCath; Medikit, Tokyo, Japan). Mechanical aspiration embolectomy was performed using a 6-Fr Indigo thrombectomy system (CAT6; Penumbra, Alameda, CA, USA). For manual aspiration, a 20-mL negative pressure syringe with male luer lock (VacLok; Merit Medical, South Jordan, UT, USA) was used to create suction while the catheter was slowly withdrawn through the guiding sheath for removal of the clot from the vessel. For mechanical aspiration, the Indigo System Separator 6 (SEP6; Penumbra) was used through the aspiration catheter to enhance clot removal. Before 2024, manual aspiration embolectomy was performed in all cases. Since its approval for ASMAO treatment at our institution in 2024, the Indigo System has become the standard tool for mechanical aspiration embolectomy and has been applied in all subsequent cases requiring aspiration embolectomy.

A multiple sidehole infusion catheter (Fountain Infusion System; Merit Medical, South Jordan, UT, USA) was advanced over a 0.035-inch guidewire (Radifocus) into the thromboembolus in the affected SMA. Then, forced periodic infusion of urokinase was performed (Mochida Pharmaceutical, Tokyo, Japan). For infusion, 1 vial of urokinase (60,000 units) was dissolved in 20 mL of saline solution and administered over a duration of 5 min. The maximum amount of urokinase used per procedure was set at 240,000 units. In cases of incomplete EVT with residual clot, a heparin-coated catheter (Anthron; Toray Medical, Tokyo, Japan) was left in SMA, and adjunctive continuous local thrombolysis with urokinase (240,000 units/day) was performed via an automated pump between EVT sessions.

Stent implantation was performed using a self-expandable bare-metal stent (LIFESTENT SOLO; BD, Franklin Lakes, NJ, USA, or SMART control; Cordis, Santa Clara, CA, USA). The guiding sheath was advanced to the distal side of the point of stent implantation, and a 0.035-inch stiff guidewire (Amplatz Super Stiff) was used for stent delivery. Stent diameters approximately 1 mm larger than the target vessel diameter were selected.

Balloon angioplasty was performed using a balloon catheter (Mustang; Boston Scientific, Natick, MA, USA). A 0.035-inch guidewire (Radifocus or Amplatz Super Stiff) was used for balloon delivery. Balloon diameters were selected to match the target vessel size.

Aspiration embolectomy was typically used for larger vessels, such as those with proximal or middle thrombus. Local thrombolysis was primarily applied for smaller vessels, particularly those with peripheral thrombus. Antegrade stenting was indicated for larger vessels, including those with proximal thrombus. Balloon angioplasty was reserved for cases where all other endovascular techniques were ineffective, as it carries a high risk of distal embolization.

In this study, all EVT procedures were performed or supervised by the endovascular treatment team at the same institution, which always included at least one board-certified interventional radiologist. As a general rule, the procedures were conducted according to the algorithm based on thrombus location; however, certain steps were omitted or the sequence was modified at the clinical discretion of the operator. Such deviations were rare, and the overall consistency of algorithm application in this study was high.

### Initial EVT strategy depends on the thrombus locations

**Fig. 2:**
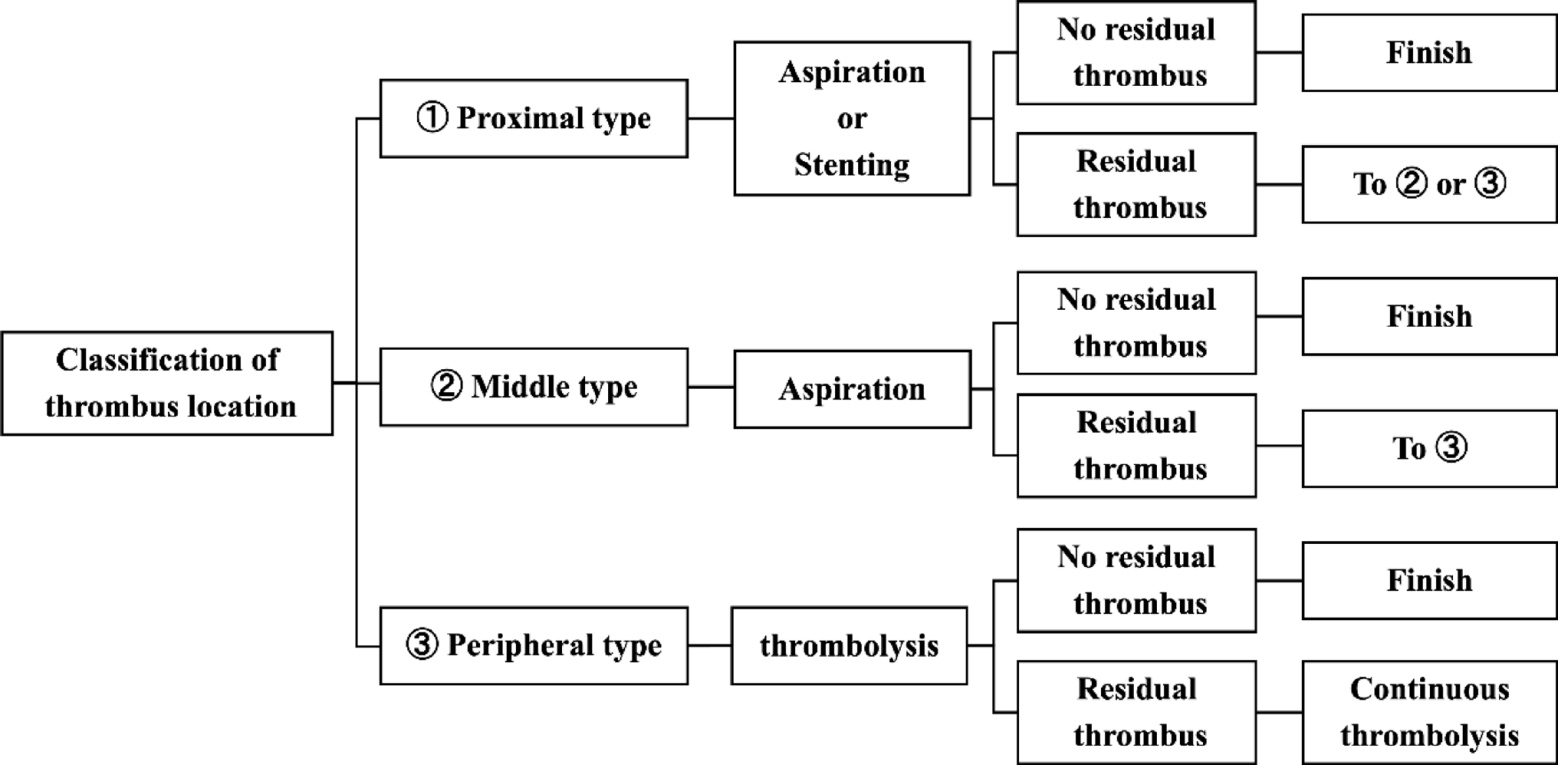
Endovascular therapy (EVT) strategy based on thrombus locations

The initial EVT strategy employed at our institution is illustrated in Fig. 2. The strategy was determined based on thrombus location. For proximal thrombus, aspiration embolectomy or antegrade stenting was performed as the initial EVT. For occlusions confined to the proximal region, antegrade stenting was initially performed. For other cases, aspiration embolectomy was initially performed. For middle thrombus, aspiration embolectomy was performed as the initial EVT. For peripheral thrombus, local thrombolysis was performed as the initial EVT. In cases with thrombi present in multiple segments, the initial EVT strategy was based on the most proximal thrombus. If residual thrombus persisted after the initial techniques, additional techniques were employed. If the thrombus remained despite these techniques, continuous local thrombolysis was administered between EVT sessions, with subsequent sessions scheduled several days later as necessary.

### Efficacy assessment

The following data were collected for analysis: patient age, sex, cause of ASMAO, time from symptom onset, synchronous embolization of other arteries, classification of thrombus location, follow-up duration, type of endovascular techniques, technical success, procedure-related major adverse events, bowel necrosis, bowel resection (including length of bowel resection), all-cause 30-day mortality, and ASMAO-related 30-day mortality. The cause of ASMAO was classified as embolism, atherothrombosis, or unknown. Cardiac embolism was diagnosed when patients had atrial fibrillation with an abrupt SMA occlusion pattern on CT or angiography, without significant atherosclerosis. Aortic embolism was diagnosed when CT showed an aortic mural thrombus or plaque corresponding to the SMA occlusion site, with no other embolic source. Atherothrombosis was defined as occlusion associated with marked atherosclerotic changes in SMA. Cases not meeting these criteria were classified as unknown. Technical success was defined as the achievement of reperfusion of blood flow in the primary occluded SMA segment targeted by EVT, as confirmed on completion angiography performed during the final EVT procedure. Technical success was considered achieved even if residual thrombus persisted in distal or peripheral branches while flow was restored in the proximal/middle SMA. Adverse events were classified according to the Society of Interventional Radiology guidelines [19].

### Statistical analysis

Continuous variables are presented as means ± standard deviations or interquartile range, while categorical variables are expressed as numbers and percentages. Survival curves were generated using the Kaplan–Meier method with the log-rank test. For the sensitivity analysis involving the three groups, categorical outcomes were compared using the chi‐square test. Factors influencing ASMAO-related 30-day mortality (age, sex, time from symptom onset, thrombus location type, and bowel necrosis) were analyzed using univariate analysis with the log-rank test. All statistical analyses were conducted using SPSS for Windows version 28 (IBM Corp., Armonk, NY, USA). A *P* value < 0.05 was considered statistically significant.

## Results

**Fig. 3:**
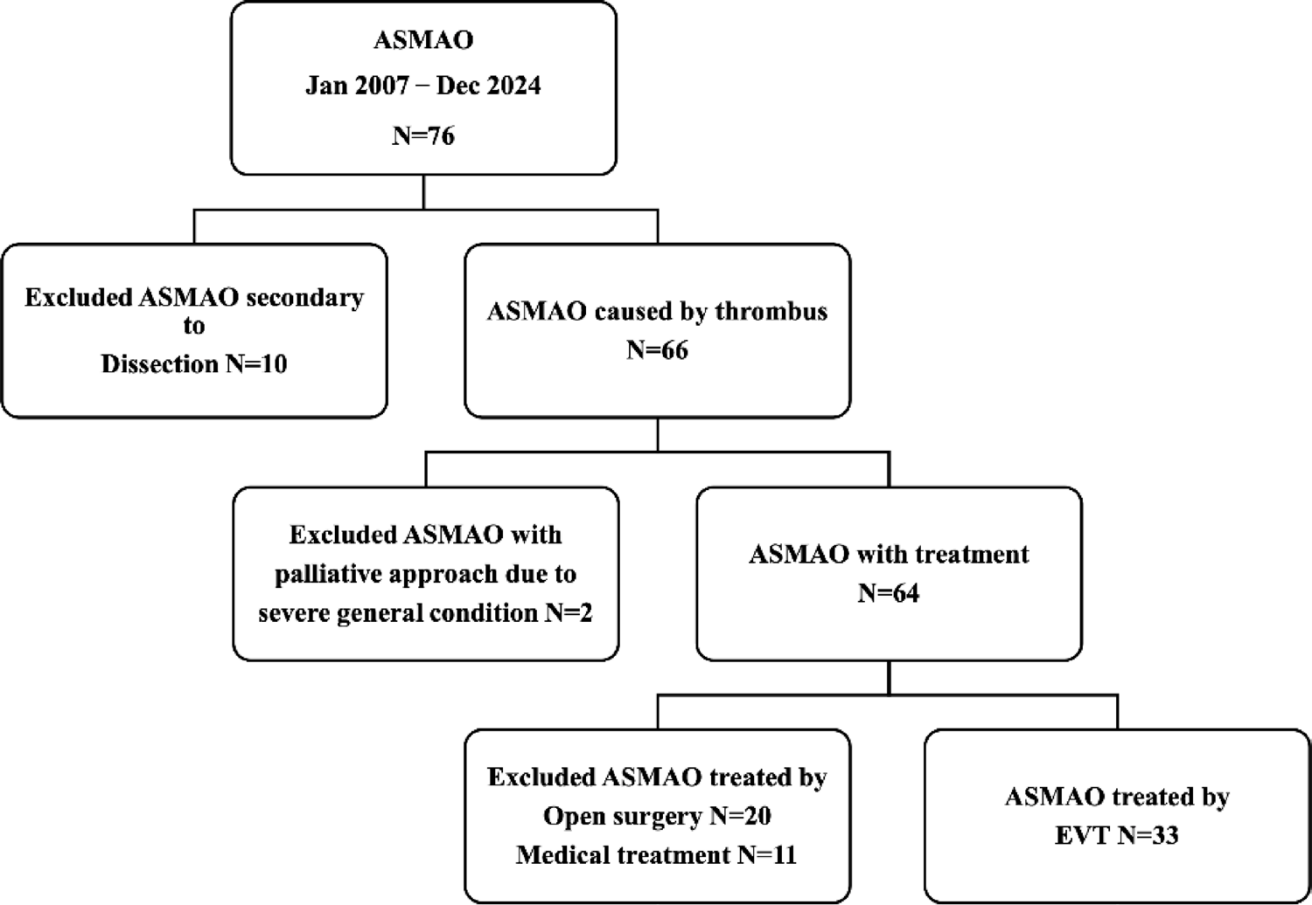
Flowchart of patient selection. The flowchart illustrates the patient selection process used in this study for the 76 patients diagnosed with ASMAO. *ASMAO* acute superior mesenteric artery occlusion, *EVT* endovascular therapy

The flowchart of patient selection is shown in Fig. 3. In total, 76 consecutive patients were diagnosed with ASMAO (45 male and 31 female patients). Ten patients with ASMAO secondary to dissection were excluded. Additionally, two patients were excluded because they received palliative care owing to severe general conditions; an additional 31 patients were excluded because they were treated without EVT (20 with open surgery and 11 with medical treatment alone). For the 20 patients who underwent open surgery alone, bowel resection was prioritized over revascularization based on clinical judgment. Six of these patients underwent bowel resection but died without revascularization because of poor general condition. For the remaining 14 patients, revascularization was deemed unnecessary after bowel resection because no residual thrombus or only a small amount of thrombus was observed. For the 11 patients who received medical treatment alone, peritonitis was considered unlikely, and revascularization was considered unnecessary. Ultimately, 33 consecutive patients who underwent EVT for ASMAO were included in this study. Among the 33 patients, 21 received exploratory laparotomy during the study period. From these patients, seven received laparotomy prior to EVT, and three of them required bowel resection followed by EVT. These patients were included in the final cohort of 33 EVT cases.

### Patient characteristics

Patient characteristics are summarized in Table 1. The mean age was 76.0 ± 8.9 years (range 55–90 years), with 20 male and 13 female patients. The causes of ASMAO were as follows: embolism, 81.8% (n = 27); atherothrombosis, 12.1% (n = 4); and unknown, 6.1% (n = 2). Among the 27 cases of embolism, 96.3% (n = 26) involved embolism with a cardiac origin, all caused by atrial fibrillation. The remaining case involved embolism from an aortic thrombus. The time from symptom onset was distributed as follows: ≤ 6 h, 21.2% (n = 7); 6–24 h, 24.2% (n = 8); and ≥ 24 h, 54.5% (n = 18). Synchronous embolization was observed in 21.2% (n = 7), including cases involving the brain (n = 3), lower limb (n = 2), and kidney (n = 2). Thrombus location were as follows: Type 1, 6.1% (n = 2); Type 2, 15.2% (n = 5); Type 3, 6.1% (n = 2); Type 4, 6.1% (n = 2); Type 5, 48.5% (n = 16); and Type 6, 18.2% (n = 6). The *median* follow-up duration was 64 days (IQR 24–244 days; mean, 518 ± 1017 days).

**Table 1:** Patient clinical characteristics

Patients	33
Age (years, mean ± SD)	76.0 ± 8.9
Sex (male/female)	20/13
Cause, No. (%)	
Embolism	27 (81.8)
Cardiac	26 (78.8)
Aortic	1 (3.0)
Athero-thrombosis	4 (12.1)
Unknown	2 (6.1)
Time from onset, no. (%)	
≤ 6 h	7 (21.2)
6–24 h	8 (24.2)
≥ 24 h	18 (54.5)
Synchronous emboli, no. (%)	7 (21.2)
Brain	3 (9.1)
Lower limb	2 (6.1)
Kidney	2 (6.1)
Classification of thrombus location, no. (%)	
Type 1 (Proximal type)	2 (6.1)
Type 2 (Middle type)	5 (15.2)
Type 3 (Peripheral type)	2 (6.1)
Type 4 (Proximal ~ middle type)	2 (6.1)
Type 5 (Middle ~ peripheral type)	16 (48.5)
Type 6 (Proximal ~ peripheral type)	6 (18.2)
Follow-up duration (days, median [IQR]; mean ± SD)	64 [24–244]; 518 ± 1017

*IQR* interquartile range, *SD* standard deviation

### Technical and clinical outcomes

**Fig. 4:**
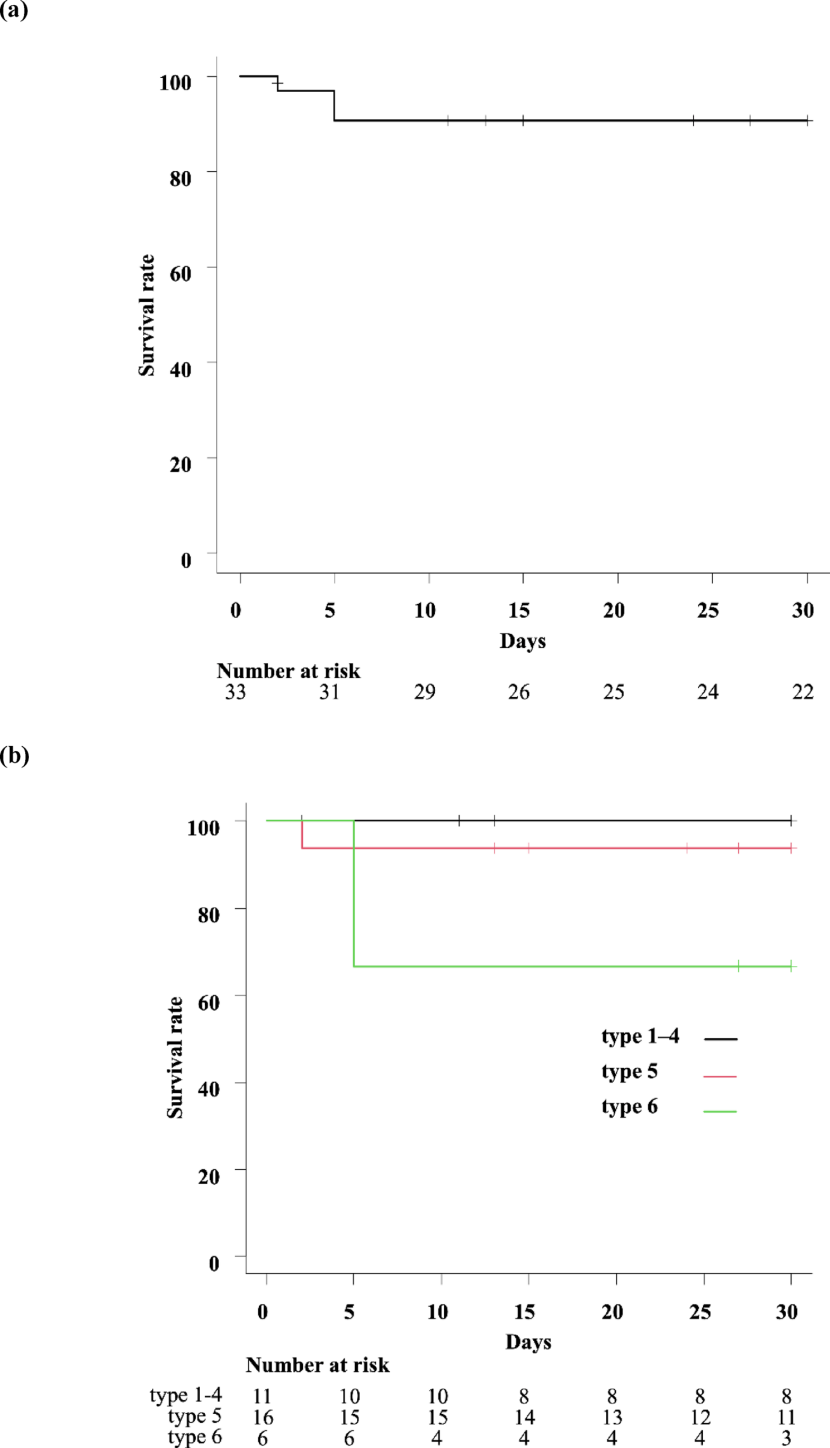
ASMAO-related 30-day survival rates. **a** ASMAO-related 30-day survival rate in the entire cohort, **b** ASMAO-related 30-day survival rate based on thrombus location. *ASMAO* acute superior mesenteric artery occlusion

The procedural details and clinical outcomes are summarized in Tables 2 and 3. Among the 33 patients, 45.5% (n = 15) received a single EVT technique: aspiration embolectomy, 21.2% (n = 7); local thrombolysis, 6.1% (n = 2); and stenting, 18.2% (n = 6). Multiple EVT techniques were used for 54.5% patients (n = 18). The combinations of multiple EVT techniques are shown in Table 2. The technical success rate was 100%. Procedure-related major adverse events occurred in 15.2% patients (n = 5). Arterial injury due to aspiration embolectomy occurred in 12.1% patients (n = 4, bleeding in three and dissection in one), and distal embolization due to balloon angioplasty occurred in 3.0% patients (n = 1). Bowel necrosis occurred in 36.4% patients (n = 12), with 33.3% (n = 11) requiring necrotic bowel resection. One patient did not undergo laparotomy despite bowel necrosis because of a severe general condition. All-cause 30-day death occurred in five patients, with a mortality rate of 15.8%. Of these, three patients died from ASMAO-related causes, while two died from other causes (heart failure in one, unknown in one). According to the Kaplan–Meier method, the ASMAO-related 30-day mortality rate was 9.3% (n = 3) in the entire cohort (Fig. 4a). Details of bowel necrosis and ASMAO-related deaths are presented in Table 4. All patients with bowel necrosis had the peripheral thrombus type (Type 5 in 10, Type 6 in two). Extensive thrombus involving the peripheral region (types 5 and 6) was significantly associated with bowel necrosis, compared with the other types (types 1–4; *P* < 0.05). Five of the nine surviving patients with bowel resection developed short bowel syndrome. All ASMAO-related 30-day deaths occurred in patients with type 5 or 6 thrombus location, which was associated with extensive bowel necrosis. Based on thrombus location, the ASMAO-related 30-day mortality rates were 0% for types 1–4, 6.2% (n = 1) for type 5, and 33.3% (n = 2) for type 6, as determined by the Kaplan–Meier method (Fig. 4b). When the six types were collapsed into three conventional groups (proximal, n = 10; middle, n = 21; distal n = 2), bowel necrosis was observed in 20.0% (n = 2), 47.6% (n = 10), and 0% cases, respectively (*P* = 0.18). ASMAO-related 30-day mortality was 20.0% (n = 2), 4.8% (n = 1), and 0%, respectively (*P* = 0.35). According to univariate analysis using the log-rank test regarding factors that might have influenced the ASMAO-related 30-day mortality, bowel necrosis and type 6 thrombus location were identified as risk factors for ASMAO-related death (*P* = 0.02 and *P* = 0.03, respectively). On the other hand, age, sex, and time from symptom onset were not significantly associated with ASMAO-related 30-day mortality.

**Table 2:** Procedural details

	No. (%)
Single EVT technique	15 (45.5)
Aspiration embolectomy	7 (21.2)
Local thrombolysis	2 (6.1)
Antegrade stenting	6 (18.2)
Multiple EVT techniques	18 (54.5)
Aspiration embolectomy + Local thrombolysis	12 (36.4)
Aspiration embolectomy + Antegrade stenting	2 (6.1)
Aspiration embolectomy + Local thrombolysis + Balloon angioplasty	2 (6.1)
Local thrombolysis + Antegrade stenting	1 (3.0)
Aspiration embolectomy + Antegrade stenting + Balloon angioplasty	1 (3.0)
EVT techniques	
Aspiration embolectomy	24 (72.7)
Local thrombolysis	17 (51.5)
Continuous local thrombolysis	11 (33.3)
Antegrade stenting	10 (30.3)
Balloon angioplasty	3 (9.1)

*EVT* endovascular therapy

**Table 3:** Clinical outcomes

Technical success	33 (100)
Procedure-related adverse events	5 (15.2)
Bowel necrosis	12 (36.4)
Bowel resection	11 (33.3)
All-cause 30-day mortality	5 (15.8)
ASMAO-related 30-day mortality	3 (9.3)

*ASMAO* acute superior mesenteric artery occlusion

**Table 4:** Details of bowel necrosis and ASMAO-related mortality

Case	Age	Sex	Classification of thrombus location	Bowel necrosis	Bowel resection range	Short bowel syndrome	ASMAO-related 30-days mortality
1	77	M	Type 5	Yes	130 cm of small intestine	No	No
2	68	M	Type 5	Yes	50 cm of jejunum	No	No
3	87	M	Type 5	Yes	140 cm of ileum	No	No
4	83	M	Type 5	Yes	130 cm of ileum	No	No
5	85	M	Type 5	Yes	Total small intestine + A-colon	Yes	No
6	66	F	Type 5	Yes	400 cm of small intestine + A-colon	Yes	No
7	90	F	Type 5	Yes	100 cm of jejunum	Yes	No
8	71	M	Type 5	Yes	130 cm of small intestine	Yes	No
9	79	F	Type 5	Yes	500 cm of small intestine	Yes	No
10	76	M	Type 5	Yes	Total ileum + A-colon	–	Yes (POD 2)
11	76	F	Type 6	Yes	Total small intestine + S-colon	–	Yes (POD 5)
12	79	F	Type 6	Yes	– (unable to perform bowel resection)	–	Yes (POD 5)

*ASMAO* acute superior mesenteric artery occlusion, *A-colon* ascending colon, *F* female, *M* male, *POD* postoperative day, *S-colon* sigmoid colon

## Discussion

In the present study, an EVT strategy based on thrombus location for ASMAO demonstrated *favorable* technical success rates (100%) and low ASMAO-related 30-day mortality (9.3%) compared with those of previous reports (technical success rates: 88–91.9%, 30-day mortality rate: 27–32%) [8, 20–22]. The EVT strategy employed in this study involved selecting the initial EVT techniques based on thrombus location.

For proximal types of thrombi, stent placement or aspiration embolectomy were applied owing to their suitability for relatively larger vessels. Aspiration embolectomy enabled rapid reperfusion while minimizing the risk of distal embolization [23, 24]. However, aspiration embolectomy was associated with a risk of vessel injury owing to the use of large-diameter catheters.

Stent placement compresses the thrombus and fixes it to the vessel wall, thereby preventing thrombus dislodgement. This technique can rapidly achieve revascularization with a lower risk of distal embolization and bleeding [24]. In contrast, balloon angioplasty, although similar to stent placement, may elevate the risk of distal embolization. However, additional comparative data or more detailed outcome analyses are required to substantiate the theoretical advantages of stent placement [24].

For peripheral thrombi, local thrombolysis was used because smaller vessels present challenges for aspiration embolectomy and stent placement. Although thrombolysis is effective for peripheral thrombi, it carries risks such as hemorrhage and thrombus migration, particularly with thrombolytic agents such as urokinase [23]. Notably, no hemorrhagic events due to urokinase occurred in this study. This could be attributed to the EVT strategy, which prioritized aspiration embolectomy or stenting for proximal and middle thrombi, reducing the overall requirement for urokinase. Furthermore, continuous local thrombolysis for residual thrombi complemented the primary treatment, likely contributing to improved outcomes. In this study, the combination of continuous thrombolysis with other techniques appears to have mitigated the associated risks effectively.

Another potential factor contributing to the low mortality rate was the use of a hybrid treatment strategy involving resection of the necrotic bowel at the appropriate time, in addition to an appropriate EVT treatment strategy based on thrombus location. Indeed, hybrid approaches to ASMAO demonstrated superior outcomes compared with those of traditional open or endovascular approaches [25, 26].

All patients with bowel necrosis had peripheral type thrombi (Type 5 or 6), and all ASMAO-related deaths were associated with extensive bowel necrosis. These findings suggest that peripheral thrombus with extensive bowel necrosis may serve as a prognostic factor for poor outcomes. Indeed, the ASMAO-related 30-day mortality rates in Types 5 and 6 tended to be higher than the other types. Furthermore, for ASMAO-related 30-day mortality, bowel necrosis and Type 6 thrombus locations were identified as risk factors. In previous literature, studies directly evaluating the anatomical location of SMA thrombi as a primary determinant of bowel necrosis are limited [16, 27–29]. Types 1, 2, and 4 (no peripheral thrombus) and Type 3 (limited peripheral involvement), as classified in the present study, are physiologically less prone to necrosis because collateral perfusion can preserve flow. In contrast, Types 5 and 6 represent extensive, peripherally predominant, multibranch occlusions that interrupt longitudinal flow along the marginal arcade and constrain collateral recruitment, thus increasing the risk of irreversible ischemia. The higher incidence of necrosis observed in patients with Types 5 and 6 in our study is therefore consistent with this pathophysiological model. Nevertheless, these findings should be interpreted as associations rather than proof of causality. Potential confounders such as the time from symptom onset to reperfusion, thrombus etiology, baseline collateral robustness, and hemodynamic status may influence both thrombus distribution and outcomes. Our results indicate that the prognosis for ASMAO with extensive bowel necrosis remains poor, highlighting the importance of optimizing treatment strategies and promoting early diagnosis and intervention. For the analysis of ASMAO-related 30-day mortality, we selected key variables based on findings from previous studies and clinical relevance and examined them using univariate analysis. However, the number of cases was limited, and we considered the statistical power insufficient to perform a reliable multivariate analysis.

Although the conventional three-tier classification for thrombus location (proximal, middle, distal) is simple and intuitive [16], it can become ambiguous when the thrombus spans multiple arterial segments. Our classification into six thrombus patterns incorporates not only anatomical location but also the thrombus extent, approximating the thrombus burden. Consideration of both location and burden in the present study provided a more granular stratification of the risk of bowel necrosis and ASMAO-related 30-day mortality, and this may aid real-time EVT selection, prognostication, and treatment planning.

This study has several limitations. First, it was a retrospective, single-arm study, with an overall small sample size, a very small number of cases for some categories, sample heterogeneity, and a long recruitment period. Second, selection of EVT techniques was not randomized, and this may have introduced bias in EVT selection. Third, only cases treated with EVT were included, and those treated exclusively with open surgery or medical treatment alone were excluded. Therefore, extremely severe or very mild cases of ASMAO were possibly excluded, and the present findings may not fully represent the overall outcomes of ASMAO. Fourth, vessel injuries were observed when large-bore catheters were used. While catheter size and technical expertise may have contributed to these events, the small number of cases precludes definitive conclusions, and the involvement of other factors cannot be excluded. Another limitation is that the Indigo System was introduced only in 2024; this may have influenced the outcomes in the later part of the study period. This temporal bias should be taken into account when interpreting our results. Fifth, addressing poor prognostic factors is needed to improve treatments for peripheral thrombus, particularly Type 6. Potential approaches include introducing more effective devices and technologies or establishing early diagnostic markers. Although urokinase was the primary thrombolytic agent in this study, its use has been restricted because of the COVID-19 pandemic. Finally, although this study focused on 30-day outcomes, evaluating long-term prognosis, including re-occlusion and survival rates, is necessary.

In conclusion, tailored endovascular strategies based on thrombus location may contribute to favorable clinical outcomes in patients with ASMAO. Future studies should employ large-scale, longitudinal, multicenter, randomized controlled study designs to further validate these findings.

## References

[1] Björck M, Koelemay M, Acosta S, Bastos Goncalves F, Kölbel T, Kolkman JJ, et al. Editor’s choice—management of the diseases of mesenteric arteries and veins: clinical practice guidelines of the European Society of Vascular Surgery (ESVS). Eur J Vasc Endovasc Surg. 2017;53:460–510. 10.1016/j.ejvs.2017.01.010. 28359440 10.1016/j.ejvs.2017.01.010

[2] Beaulieu RJ, Arnaoutakis KD, Abularrage CJ, Efron DT, Schneider E, Black JH 3rd. Comparison of open and endovascular treatment of acute mesenteric ischemia. J Vasc Surg. 2014;59(1):159–64. 10.1016/j.jvs.2013.06.084. 24199769 10.1016/j.jvs.2013.06.084

[3] Ryer EJ, Kalra M, Oderich GS, Duncan AA, Gloviczki P, Cha S, et al. Revascularization for acute mesenteric ischemia. J Vasc Surg. 2012;55(6):1682–9. 10.1016/j.jvs.2011.12.017. 22503176 10.1016/j.jvs.2011.12.017

[4] Arthurs ZM, Titus J, Bannazadeh M, Eagleton MJ, Srivastava S, Sarac TP, et al. A comparison of endovascular revascularization with traditional therapy for the treatment of acute mesenteric ischemia. J Vasc Surg. 2011;53:698–704. 10.1016/j.jvs.2010.09.049. (**discussion 704 [Discussion]**). 21236616 10.1016/j.jvs.2010.09.049

[5] Block TA, Acosta S, Björck M. Endovascular and open surgery for acute occlusion of the superior mesenteric artery. J Vasc Surg. 2010;52(4):959–66. 10.1016/j.jvs.2010.05.084. 20620006 10.1016/j.jvs.2010.05.084

[6] Schermerhorn ML, Giles KA, Hamdan AD, Wyers MC, Pomposelli FB. Mesenteric revascularization: management and outcomes in the United States, 1988–2006. J Vasc Surg. 2009;50:341-8.e1. 10.1016/j.jvs.2009.03.004. 19372025 10.1016/j.jvs.2009.03.004 PMC2716426

[7] Sasaki K, Okada T, Yamaguchi M, Ozaki M, Okamoto Y, Umeno A, et al. Interventional radiology in treating acute mesenteric arterial occlusion: a narrative review. Interv Radiol. 2025;10:e20240018. 10.22575/interventionalradiology.2024-0018. 10.22575/interventionalradiology.2024-0018 PMC12078031 40384917

[8] Raupach J, Lojik M, Chovanec V, Renc O, Strýček M, Dvořák P, et al. Endovascular management of acute embolic occlusion of the superior mesenteric artery: a 12-year single-centre experience. Cardiovasc Intervent Radiol. 2016;39:195–203. 10.1007/s00270-015-1156-6. 26202388 10.1007/s00270-015-1156-6

[9] Heiss P, Loewenhardt B, Manke C, Hellinger A, Dietl KH, Schlitt HJ, et al. Primary percutaneous aspiration and thrombolysis for the treatment of acute embolic superior mesenteric artery occlusion. Eur Radiol. 2010;20:2948–58. 10.1007/s00330-010-1859-7. 20563813 10.1007/s00330-010-1859-7

[10] Acosta S, Sonesson B, Resch T. Endovascular therapeutic approaches for acute superior mesenteric artery occlusion. Cardiovasc Intervent Radiol. 2009;32:896–905. 10.1007/s00270-009-9559-x. 19365685 10.1007/s00270-009-9559-x

[11] Wong PF, Gilliam AD, Kumar S, Shenfine J, O’Dair GN, Leaper DJ. Antibiotic regimens for secondary peritonitis of gastrointestinal origin in adults. Cochrane Database Syst Rev. 2005;2005:CD004539. 10.1002/14651858.CD004539.pub2. 15846719 10.1002/14651858.CD004539.pub2 PMC11297476

[12] Yanar F, Agcaoglu O, Sarici IS, Sivrikoz E, Ucar A, Yanar H, et al. Local thrombolytic therapy in acute mesenteric ischemia. World J Emerg Surg. 2013;8:8. 10.1186/1749-7922-8-8. 23394456 10.1186/1749-7922-8-8 PMC3626770

[13] Björnsson S, Björck M, Block T, Resch T, Acosta S. Thrombolysis for acute occlusion of the superior mesenteric artery. J Vasc Surg. 2011;54(6):1734–42. 10.1016/j.jvs.2011.07.054. 21889287 10.1016/j.jvs.2011.07.054

[14] Freitas B, Bausback Y, Schuster J, Ulrich M, Bräunlich S, Schmidt A, et al. Thrombectomy devices in the treatment of acute mesenteric ischemia: initial single-center experience. Ann Vasc Surg. 2018;51:124–31. 10.1016/j.avsg.2017.11.041. 29455017 10.1016/j.avsg.2017.11.041

[15] Gries JJ, Virk HUH, Chen B, Sakamoto T, Alam M, Krittanawong C. Advancements in revascularization strategies for acute mesenteric ischemia: a comprehensive review. J Clin Med. 2024;13(2):570. 10.3390/jcm13020570. 38276076 10.3390/jcm13020570 PMC10816895

[16] Tual A, Garzelli L, Nuzzo A, Corcos O, Castier Y, Ben Abdallah I, et al. Strengthening the description of superior mesenteric artery occlusions in acute mesenteric ischaemia: proposition for an anatomical classification. Eur J Vasc Endovasc Surg. 2023;65(6):802–8. 10.1016/j.ejvs.2023.01.041. 36736617 10.1016/j.ejvs.2023.01.041

[17] Najdawi M, Garzelli L, Nuzzo A, Huguet A, Raynaud L, Paulatto L, et al. Endovascular revascularization of acute arterial mesenteric ischemia: report of a 3-year experience from an intestinal stroke center unit. Eur Radiol. 2022;32:5606–15. 10.1007/s00330-022-08660-3. 35258671 10.1007/s00330-022-08660-3

[18] Jia Z, Jiang G, Tian F, Zhao J, Li S, Wang K, et al. Early endovascular treatment of superior mesenteric occlusion secondary to thromboemboli. Eur J Vasc Endovasc Surg. 2014;47(2):196–203. 10.1016/j.ejvs.2013.09.025. 24183620 10.1016/j.ejvs.2013.09.025

[19] Baerlocher MO, Nikolic B, Sze DY. Adverse event classification: clarification and validation of the Society of Interventional Radiology specialty-specific system. J Vasc Interv Radiol. 2023;34(1):1–3. 10.1016/j.jvir.2022.10.011. 36244632 10.1016/j.jvir.2022.10.011

[20] Pengermä P, Venesmaa S, Karjalainen J, Ukkonen M, Saari P, Kärkkäinen JM. Long-term outcome after implementation of endovascular-first strategy to treat acute mesenteric ischemia. J Vasc Surg. 2023;78(6):1524–30. 10.1016/j.jvs.2023.08.100. 37586616 10.1016/j.jvs.2023.08.100

[21] Altintas Ü, Lawaetz M, de la Motte L, Riazi H, Lönn L, Lindh M, et al. Endovascular treatment of chronic and acute on chronic mesenteric ischaemia: results from a national cohort of 245 cases. Eur J Vasc Endovasc Surg. 2021;61:603–11. 10.1016/j.ejvs.2021.01.003. 33589326 10.1016/j.ejvs.2021.01.003

[22] Kärkkäinen JM, Lehtimäki TT, Saari P, Hartikainen J, Rantanen T, Paajanen H, et al. Endovascular therapy as a primary revascularization modality in acute mesenteric ischemia. Cardiovasc Intervent Radiol. 2015;38:1119–29. 10.1007/s00270-015-1064-9. 25737456 10.1007/s00270-015-1064-9

[23] Ueda T, Murata S, Miki I, Yasui D, Sugihara F, Tajima H, et al. Endovascular treatment strategy using catheter-directed thrombolysis, percutaneous aspiration thromboembolectomy, and angioplasty for acute upper limb ischemia. Cardiovasc Intervent Radiol. 2017;40:978–86. 10.1007/s00270-017-1599-z. 28184959 10.1007/s00270-017-1599-z

[24] Ueda T, Tajima H, Murata S, Saito H, Yasui D, Sugihara F, et al. A comparison of outcomes based on vessel type (Native artery vs. bypass Graft) and artery Location (Below-Knee artery vs. non-Below-Knee artery) using a combination of multiple endovascular techniques for acute lower limb ischemia. Ann Vasc Surg. 2021;75:205–16. 10.1016/j.avsg.2021.02.023. 33819584 10.1016/j.avsg.2021.02.023

[25] Salsano G, Salsano A, Sportelli E, Petrocelli F, Dahmane M, Spinella G, et al. What is the best revascularization strategy for acute occlusive arterial mesenteric ischemia: systematic review and meta-analysis. Cardiovasc Intervent Radiol. 2018;41:27–36. 10.1007/s00270-017-1749-3. 28752257 10.1007/s00270-017-1749-3

[26] Zientara A, Domenghino AR, Schwegler I, Bruijnen H, Schnider A, Weber M, et al. Interdisciplinary approach in emergency revascularization and treatment for acute mesenteric ischemia. BMC Surg. 2021;21:89. 10.1186/s12893-021-01102-9. 33602217 10.1186/s12893-021-01102-9 PMC7890998

[27] Bala M, Catena F, Kashuk J, De Simone B, Gomes CA, Weber D, et al. Acute mesenteric ischemia: updated guidelines of the World Society of Emergency Surgery. World J Emerg Surg. 2022;17:54. 10.1186/s13017-022-00443-x. 36261857 10.1186/s13017-022-00443-x PMC9580452

[28] Hawthorn BR, Ratnam LA. Acute mesenteric ischaemia: imaging and intervention. Clin Radiol. 2020;75:398.e19-28.e28. 10.1016/j.crad.2019.06.001. 31320112 10.1016/j.crad.2019.06.001

[29] Olson MC, Fletcher JG, Nagpal P, Froemming AT, Khandelwal A. Mesenteric ischemia: what the radiologist needs to know. Cardiovasc Diagn Ther. 2019;9(1):S74-87. 10.21037/cdt.2018.09.06. 31559155 10.21037/cdt.2018.09.06 PMC6732105

